# PGGait: Gait Recognition Based on Millimeter-Wave Radar Spatio-Temporal Sensing of Multidimensional Point Clouds

**DOI:** 10.3390/s24010142

**Published:** 2023-12-27

**Authors:** Xiaochao Dang, Yangyang Tang, Zhanjun Hao, Yifei Gao, Kai Fan, Yue Wang

**Affiliations:** 1 College of Computer Science and Engineering, Northwest Normal University, Lanzhou 730070, China; 2022222253@nwnu.edu.cn (Y.T.); haozhj@nwnu.edu.cn (Z.H.); 2022212162@nwnu.edu.cn (Y.G.); 2022222251@nwnu.edu.cn (K.F.); 2022222225@nwnu.edu.cn (Y.W.); 2 Gansu Province Internet of Things Engineering Research Center, Lanzhou 730070, China

**Keywords:** gait recognition, identity verification, millimeter-wave radar, point cloud, neural network

## Abstract

Gait recognition, crucial in biometrics and behavioral analytics, has applications in human–computer interaction, identity verification, and health monitoring. Traditional sensors face limitations in complex or poorly lit settings. RF-based approaches, particularly millimeter-wave technology, are gaining traction for their privacy, insensitivity to light conditions, and high resolution in wireless sensing applications. In this paper, we propose a gait recognition system called Multidimensional Point Cloud Gait Recognition (PGGait). The system uses commercial millimeter-wave radar to extract high-quality point clouds through a specially designed preprocessing pipeline. This is followed by spatial clustering algorithms to separate users and perform target tracking. Simultaneously, we enhance the original point cloud data by increasing velocity and signal-to-noise ratio, forming the input of multidimensional point clouds. Finally, the system inputs the point cloud data into a neural network to extract spatial and temporal features for user identification. We implemented the PGGait system using a commercially available 77 GHz millimeter-wave radar and conducted comprehensive testing to validate its performance. Experimental results demonstrate that PGGait achieves up to 96.75% accuracy in recognizing single-user radial paths and exceeds 94.30% recognition accuracy in the two-person case. This research provides an efficient and feasible solution for user gait recognition with various applications.

## 1. Introduction

Amidst the rapid evolution of the Internet of Things (IoT) and pervasive computing, Cyber–physical Systems (CPSs) have emerged. These systems integrate sensors, embedded technologies, and network capabilities to realize real-time sensing and control of the physical world. In this complex ecosystem, accurate user identification is crucial, supporting access control and data security and providing a solid foundation for various intelligent applications to flourish. However, early user identification systems heavily relied on complex passwords, including combinations of letters, numbers, and special characters. While individuals widely use passwords, these credentials face several challenges, including rapid forgetfulness, susceptibility to theft, and potential security breaches. Therefore, the limitations of traditional passwords have led to the widespread use of biometrics. In recent years, researchers have actively explored biometric-based user identification methods, including facial recognition [1], fingerprint recognition [2], voiceprint recognition [3], and gait recognition [4]. In this regard, gait recognition presents a unique potential as a biometric identification method. Gait patterns are unique over time and across individuals and are virtually impossible to fake. Additionally, gait recognition offers greater flexibility than other biometrics, such as fingerprinting or facial recognition, as it works at a distance and without contact. Going forward, we can expect gait recognition technology to continue flourishing, playing an essential role in intelligent interactions, personalized services, and critical areas such as physical access control and financial transaction verification. Simultaneously, one must recognize the ensuing privacy and security challenges, necessitating the implementation of appropriate measures to safeguard user data and comply with relevant regulations and legal requirements.

Research on gait recognition systems has explored various sensor modalities, including wearables [5], vision [6,7], WiFi [8,9,10], pressure sensors [11], radio frequency identification (RFID) [12], LoRa [13], and acoustic sensing [14]. However, these sensors pose several challenges in practical applications. On one hand, they usually involve additional infrastructure costs, such as installing sensor devices at specific locations, which can increase the complexity and cost of the system. On the other hand, certain sensors, like wearable devices, may cause user discomfort, reducing the overall user experience. Additionally, some approaches may require active cooperation from the user, for example, in vision systems, where the user must maintain a specific posture or wear specific clothing. Some sensors, such as WiFi and RFID, may be at risk of privacy leakage, potentially revealing the user’s location information.

In contrast to traditional sensor modalities, millimeter-wave radar is a low-power Frequency-Modulated Continuous Wave (FMCW) device with unique advantages [15,16,17]. First, the non-invasive nature of radar technology allows it to perform monitoring while maintaining privacy, as it does not involve the capture of individual images. In addition, millimeter-wave radar is insensitive to changes in lighting conditions and therefore performs more consistently in low or unstable lighting environments. In contrast, video-based gait sensing may be limited by lighting issues, limiting its reliability under certain conditions. Another advantage is the radar’s ability to penetrate, allowing it to monitor across a number of non-metallic obstacles, thus mitigating the occlusion problem that video-based sensing systems may face. In addition, millimeter-wave radar has the ability to monitor over a wide area at long distances, making it more suitable in large indoor areas, whereas video sensing systems may require more equipment to achieve the same coverage. Video sensing systems may require more equipment to adapt to different environmental conditions, increasing system complexity and maintenance costs, in contrast to radar systems which are relatively simpler. Overall, radar-based gait sensing has a number of advantages, ranging from privacy protection to system stability, making it a highly sought-after technology in the field of gait recognition.

Considering its hardware characteristics and the benefits of being non-contact and privacy-preserving, millimeter-wave radar is anticipated to offer significant advantages for facilitating gait recognition. It typically requires minimal infrastructure support (e.g., only a single millimeter-wave radar device is required), making it a promising biometric technology for many applications. To address the aforementioned challenges, we propose the PGGait (PointNet GRU Gait) user gait recognition system. First, we designed a point cloud preprocessing pipeline to obtain high-quality point cloud data. Then, we modified the original PointNet++ network structure by adding a GRU module for multiscale spatio-temporal feature extraction. Additionally, due to the relatively small number of publicly available millimeter-wave radar gait datasets, we collected gait data from nine volunteers in different scenarios to construct the original dataset. We processed the base data using spatial coordinate rotation, translation, and scaling through data enhancement methods to increase the dataset’s size. Through these efforts, we obtained a larger dataset with limited raw data and successfully trained and evaluated the model, achieving a gait recognition accuracy of up to 96.75%.

In summary, this paper presents the following contributions:

- In PGGait, we designed a point cloud preprocessing pipeline approach for denoising point cloud data captured by millimeter-wave radar. The pipeline employs a filtering method incorporating SNR threshold distribution, which is significantly improved for clustering formulas, to obtain high-quality point cloud data for individual segmentation, thus effectively reducing the negative impact caused by the environment.
- To address the suboptimal performance of existing neural networks in processing millimeter-wave radar point clouds, we propose a novel network architecture, P-GRUNet. This network incorporates a new center-of-mass sampling algorithm and introduces a GRU module for multiscale spatiotemporal feature extraction on correlated point cloud data. This innovation overcomes the limitations of PointNet++ in temporal feature processing, thereby achieving high-precision classification and identification of millimeter-wave radar point clouds.
- We comprehensively evaluated single and two-person gait recognition accuracy in several different environments. The experimental results show that our system achieves high accuracy rates of 96.75% and 94.30% for single and two-person gait recognition, respectively.

The subsequent sections of this paper are structured as follows: Section 2 delves into pertinent research within the field. Section 3 describes the theoretical basis of FMCW radar in gait recognition. Section 4 describes the design of the PGGait system in detail. Section 5 describes the experimental setup. Section 6 evaluates the experiments. Section 7 summarizes the full text.

## 2. Related Works

Gait recognition has been an important research direction in computer vision and biomedical engineering. Traditional gait recognition methods mainly rely on sensor technologies such as RGB cameras, depth cameras, and inertial sensors. However, these methods could improve performance under poor lighting conditions and occlusion. With the development of technology, wireless sensing has become an emerging field covering many attractive applications, especially in gait recognition. Among the various wireless sensing applications, radar-based systems are exactly what the PGGait project is keenly interested in. In our study, we concentrate on single and two-person scenarios, each presenting a unique set of challenges for gait recognition. Our objective is to enhance the accuracy of gait recognition across various scenarios, surpassing the performance of traditional methods in handling the complexities encountered in real-world situations.

### 2.1. WiFi-Based Approaches

WiFi-based approaches extract gait features from channel state information (CSI) to facilitate recognition. Researchers have extensively explored gait recognition based on CSI in the literature. For example, Widar [18] exploits the multipath effect of WiFi signals and performs gait recognition by analyzing the phase changes of the signals, which also enables highly sensitive monitoring of human position and movement. Wifi-id [19], on the other hand, analyzes the interference in CSI caused by user behavior to obtain unique features that can be used for individual recognition. GaitWay [20] investigates non-contact gait recognition by recognizing gait speed through wall monitoring. GaitFi [21], on the other hand, employs CSI reflected by WiFi multipath propagation to capture human gait while incorporating camera-captured video for robust gait recognition. Nevertheless, as previously highlighted, CSI exhibits high sensitivity to noise from diverse environmental directions. Consequently, the aforementioned WiFi-based methodologies restrict recognition to single users on predetermined paths. Moreover, the susceptibility of CSI to the ambient environment results in constrained generalization performance of these WiFi-based techniques to novel scenes and users. Despite researchers acknowledging deep learning as a promising avenue for WiFi cross-domain recognition, it remains essential to gather unlabeled data from new environments to make cross-domain recognition viable.

### 2.2. Radar-Based Approaches

Millimeter-wave radar stands out with its superior spatial resolution, delivering enhanced accuracy while exhibiting resistance to interference and maintaining privacy, in contrast to the methods mentioned above. Customized radars were used in early studies to extract human gait information through distance-based Doppler features [22,23,24]. However, in recent years, the availability of commercially available small millimeter-wave radar devices has increased, resulting in the emergence of multiple Doppler-feature-based gait recognition solutions. In addition, point cloud-based approaches have been proposed, extracting point cloud data from raw radar signals and then integrating these point cloud data with the help of deep learning models for individual gait recognition. Zhen et al. [25] proposed a gait recognition method that utilizes millimeter-wave sensing technology to address the challenge of coexisting with multiple people. Simultaneous multi-user recognition was achieved by analyzing the features in the millimeter-wave signals. Experimental results show that the average recognition accuracy drops from more than 90% to 88% as concurrent users go from single to multiple. This highlights the performance and limitations of the method in complex multi-person environments. In their work, Li et al. [26] extracted the spatio-temporal features of a 3D point cloud concisely and efficiently using a specially designed neural network. They constructed a new millimeter-wave radar 3D point cloud gait dataset with data enhancement of the dataset. The accuracy was also evaluated for single and multi-person gait recognition in multiple environments. The accuracy was 96.7% and 90.2%, respectively. Huang et al. [27] proposed point streaming as a novel point cloud descriptor and designed a dynamic frame sampling module to improve computational efficiency without significant performance degradation and by higher recognition performance on millimeter-wave radar datasets and advantages over traditional methods. Jiang et al. [28] proposed a human gait classification and recognition method based on millimeter-wave array radar and based on the deep learning technique to improve the residual network, a multi-channel 3D convolutional neural network was proposed to complete the hierarchical extraction and fusion of multi-dimensional features and to achieve the recognition accuracy of the gait category of more than 92.5%.

## 3. FMCW Radar Basics

When walking, each person’s bone structure, stride period and length, joint range of motion, and body proportions affect how they walk, resulting in the dynamic uniqueness of each person’s gait characteristics. The millimeter-wave radar emits a fundamentally sinusoidal signal with a continuously varying frequency. This capability enables precise measurements of an object’s distance, speed, and angle of arrival by transmitting and receiving signals reflected by obstacles in its transmission path. These measurements reflect the object’s movement and position information. Through the accurate measurement and analysis of these data, it is possible to identify and differentiate the walking patterns of different individuals, and the capture and use of these unique walking characteristics can play an essential role in a variety of applications, such as identity verification, security monitoring, and human behavior analysis.

The transmitting signal of a millimeter-wave radar continuously sends a signal with continuous linear FM whose frequency is scanned within a bandwidth B for a duration of $T_{c}$ for each linear FM signal. The slope of the scan is calculated using S=B/$T_{c}$. The radar system produces an intermediate frequency (IF) signal by combining the transmitted signal with the received signal reflected from the target object through a mixer. The specific formula is as follows:

(1) $$\;s_{IF}\left(t\right)=f_{LPF}\left\{s_{T}\left(t\right)s_{R}\left(t\right)\right\}=\frac{1}{2}A_{T}A_{R}\cdot cos2\pi \left[f_{c}\Delta t_{d}+\left(\frac{B}{T}\Delta t_{d}-\Delta f_{d}\right)t\right]$$

where $\left.s_{T}(t)\right.$ and $s_{R}(t)$ represent the transmitted and received signals, respectively, $A_{T}$ and $A_{R}$ denote the amplitudes of the transmitted and received signals, with $f_{c}$ representing the carrier’s center frequency. $\Delta t_{d}$ signifies the flight delay from the transmitted signal back to the receiver post-reflection, B is the signal bandwidth, T is the sweep period of a linear FM signal, and $\Delta f_{d}$ denotes the Doppler shift. The IF signal can then undergo processing through a frame separation procedure for subsequent applications such as frequency domain analysis.

Distance Measurement: When a user enters the detection range of a millimeter-wave radar, their body reflects a specific intermediate frequency (IF) signal. This signal can be used to determine the user’s exact location, and by performing a Fast Fourier Transform (FFT) analysis of this time-domain IF signal, a precise distance profile of the user can be obtained. Subsequently, the distance between the user and the radar can be calculated using this distance profile. Precisely, the distance can be calculated using the following formula:

(2) $$\;d=\frac{f_{IF}\times c\times T_{c}}{2\times B}=\frac{f_{IF}\times c}{2\times S}$$

where c is the speed of light, and $f_{IF}$ is the frequency of the IF signal. According to Equation (2), a precise distance measurement of the user can be obtained, and information such as precise position and step size estimation can be obtained.

Velocity Measurements: A single pulse signal is sufficient for determining the distance between the target and the radar. However, to make velocity measurements, it is necessary to leave a certain time interval ($T_{c}$) between neighboring scans and to make several scans. Each reflected pulse undergoes a Fast Fourier Transform (FFT) of the distance measurement to determine the target’s position and generate peaks with different phases. The phase difference Δ between the two scans corresponds to the displacement of the target having a velocity v. Specifically, the following relationship exists between the phase difference Δ and the velocity v.

(3) $$\;v=\frac{\lambda \Delta \phi }{4\pi T_{c}}$$

where λ is the wavelength, another FFT (i.e., the Doppler-FFT) exists to distinguish multiple users in the velocity dimension.

The Angle of Arrival Measurement: FMCW radar systems are used to determine the angle of a target’s reflected signal relative to the horizontal and vertical planes, often referred to as azimuth and elevation angles, respectively. Multi-antenna FMCW radar systems estimate the angle of arrival by analyzing the phase difference on the peak of the distance FFT caused by the distance between antennas. The relevant equations are shown below.

(4) $$\theta =\arcsin\frac{\lambda \Delta \phi }{2\pi d_{a}}$$

where $d_{a}$ is the distance between neighboring antennas, the goniometric technique allows precise determination of the target’s position in the horizontal and vertical directions.

## 4. PGGait System Design

This section provides a comprehensive overview and detailed design of the PGGait user gait recognition system, delving into the three critical components of PGGait: point cloud data collection, data preprocessing, user identification, and pattern matching.

### 4.1. System Overview

**Figure 1:**
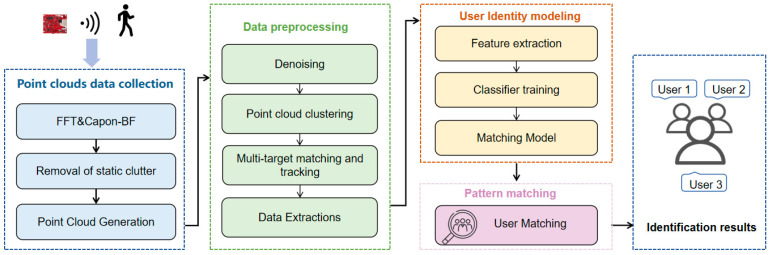
Overview of the PGGait system.

PGGait employs millimeter-wave radar to capture the gait characteristics of the human body during walking, facilitating the passive identification of multiple users. The overarching workflow of PGGait is illustrated in Figure 1, encompassing three primary components: point cloud data collection, data preprocessing, and user identification and pattern matching.

Within the module dedicated to point cloud data acquisition, we initiate the Fast Fourier Transform (FFT) to extract pertinent information on distance (d), velocity (v), and angle of arrival (θ) by Equations (2)–(4). Concurrently, we apply a Constant False Alarm Rate (CFAR) algorithm and a static cluster algorithm to mitigate interference during acquisition. These operations enable us to generate high-resolution raw point cloud data. Subsequently, the point cloud data are passed to the data preprocessing module for denoising. Then, we employ a modified DBSCAN clustering algorithm for user separation and location tracking. Finally, the multidimensional point cloud data are fed into a specially designed neural network for extracting spatial and temporal features to classify and identify multiple users.

### 4.2. Point Cloud Data Collection

**Figure 2:**
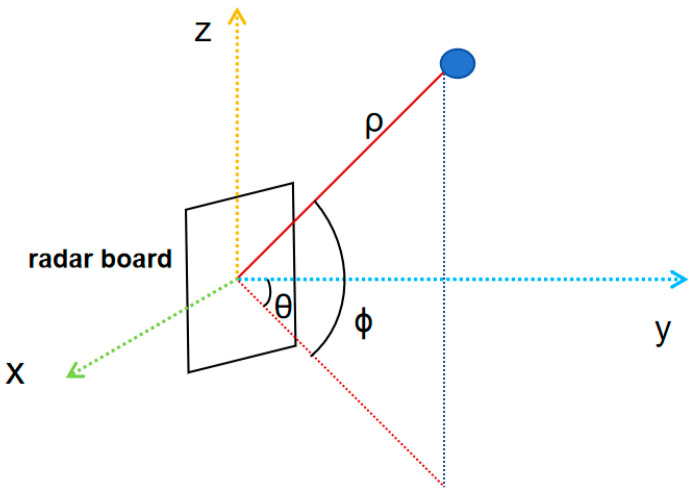
Schematic diagram of polar and Cartesian coordinates of the radar plate.

Millimeter-wave radar transmits a linear FM signal to a target and then receives and analyzes the signal reflected from the target. By measuring the signal’s time delay and phase change, we can determine the target’s location and record the reflected signal. Subsequently, point cloud data are calculated and generated from the reflected signal. The transformation of raw radar data into point cloud data involves distance FFT, velocity FFT, and angle FFT processes. Moreover, we employ Capon’s algorithm for angle of arrival (AOA) estimation, incorporating static clutter filtering and constant false alarm rate (CFAR) algorithms. During the data acquisition process, the environment becomes complex and diverse due to various disturbing factors such as noise, windows, walls, tables, chairs, passing pedestrians, and multipath effects. Therefore, we use static clutter filtering and Constant False Alarm Rate (CFAR) algorithms during data acquisition to prioritize the removal of a portion of the interfering point cloud, i.e., noise. Combining the above methods, we can transform the radar-sampled data into accurate point cloud information in a Cartesian coordinate system, as shown in Figure 2.

Converting the above ternary to the Cartesian coordinate system yields the following transformation relation for $p^{\prime }=(x,y,z)$:

(5) $$\begin{array}{c} \;\;\;\;x=\rho \times cos\left(\phi \right)\times sin\left(\theta \right)\\ \;\;\;\;y=\rho \times cos(\phi )\times cos(\theta )\\ z=\rho \times sin\left(\phi \right) \end{array}$$

where the original point coordinates are p=(ρ,∅,θ) in the ternary information, ρ is the distance Range between the point and the radar antenna, ϕ is the elevation angle Elevation of the radar antenna to the point cloud, and θ is the angle Azimuth of the point and radar connecting line with the radar facing direction in the xy-plane projection.

### 4.3. Data Preprocessing

The CFAR and static clutter algorithms [29] employed in point cloud generation prove insufficient in eliminating high-intensity points stemming from external objects. This inadequacy leads to residual noise, which persists and introduces ambiguity in the user’s data points. These are unwanted data and will have an impact on the experimental accuracy. Thus, obtaining high-quality point cloud data is a prerequisite to ensure the accuracy of the final experiment. In this context, we devise an appropriate point cloud preprocessing pipeline to acquire high-quality point cloud data, ensuring the efficacy of subsequent clustering and individual tracking processes.

**Figure 3:**
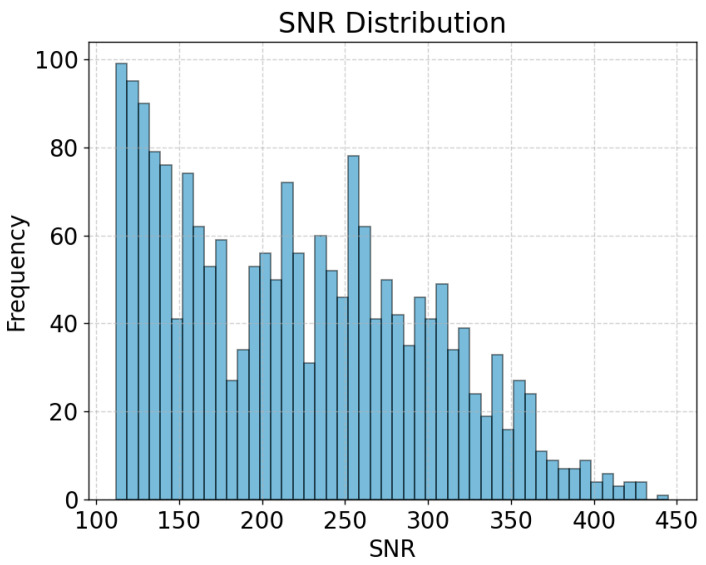
Distribution of SNR.

Denoising Phase: In the preprocessing of collected point cloud data, we employ filtering techniques, integrating the mechanism of Signal-to-Noise Ratio (SNR) distribution to determine appropriate thresholds, as illustrated in Figure 3. The motivation behind this strategy lies in leveraging the SNR distribution to identify distinctions more accurately between signals and noise. The SNR distribution provides information about the relative quality of each data point, allowing us to set thresholds based on the principle of retaining the majority of signal points while effectively filtering out noise. Additionally, we introduce a boundary threshold from real-world scenarios to further enhance the quality of the point cloud data and alleviate negative impacts caused by environmental factors. This comprehensive denoising strategy ensures the robust performance of our gait recognition system in complex environments.

**Figure 4:**
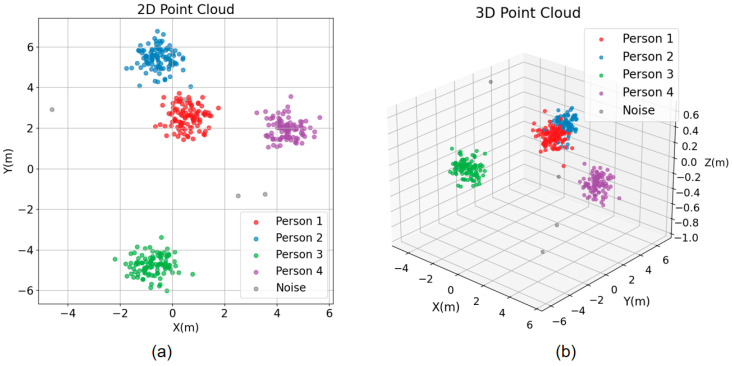
Simultaneous walking of multiple people point cloud map (**a**) 2D point cloud map (**b**) 3D point cloud map.

Point cloud clustering: PGGait selects the DBSCAN algorithm [30], a density-based spatial clustering algorithm, in the clustering phase, which can deal with noise, has good robustness, and can efficiently differentiate between anomalies and multiple target clusters. The primary strength of this algorithm lies in its ability to dispense with the pre-setting of clustering clusters. It autonomously discerns the number of point clusters, maintaining consistency with the scene. Consequently, this approach mitigates noise, thereby enhancing the accuracy of clustering. Considering the limitations of point cloud scattering in the Z-axis direction caused by the equipment, the effect of clustering using the traditional Euclidean distance could be better for the actual point cloud data clustering process. In addition, since the point cloud is relatively dense in the X-Y direction, we have improved the distance formula adopted by DBSCAN accordingly to better adapt to the distribution of the point cloud.

(6) $$dist\left(p^{i},p^{j}\right)=\sqrt{{\beta \times \left(p_{x}^{i}-p_{x}^{j}\right)}^{2}+\beta \times {\left(p_{y}^{i}-p_{y}^{j}\right)}^{2}+{\alpha \left(p_{z}^{i}-p_{z}^{j}\right)}^{2}}$$

where $p^{i}$ and $p^{j}$ are two different points in space, the results of several experiments on the values of α,β, α of 0.25, and 2β of 2.75 are relatively good. The results also show that reducing the corresponding weights for the Z-axis components improves the clustering effect. In our experiments, we set the following hyperparameters: eps (DBSCAN radius) = 0.3 and min_samples = 7. Additionally, the algorithm demonstrates improved performance when the experimental distance is maintained as large as possible, preferably greater than 0.3 m. The effect of clustering in the case of multiple people is shown in Figure 4, demonstrating the optimized performance of the algorithm.

User Tracking: In implementing user tracking, we use the Hungarian algorithm [31] to correlate the clusters that match each time interval to build their trajectories. To accomplish this, we introduce a corresponding Kalman filter for each trajectory. Expressly, we represent the target as the center of mass of a cluster of points, considered a scattering of points. To make predictions and use the Kalman filter, we design the state vectors for each trajectory as follows:

(7) $$s={\left[x,y,∆x,∆y\right]}^{T}$$

Specifically, x and y represent the horizontal and vertical coordinates of the trajectory, while ∆x and ∆y represent the change of these coordinates within one time unit, i.e., velocity information. Therefore, the state transition process can be expressed as follows:

(8) $$s_{t}=Fs_{t-1}$$

where the F transfer matrix is:

(9) $$F=\left[\begin{array}{c} 1\;\;\;\;0\;\;\;\;1\;\;\;\;0\\ 0\;\;\;\;1\;\;\;\;0\;\;\;\;1\\ 0\;\;\;\;0\;\;\;\;1\;\;\;\;0\\ 0\;\;\;\;0\;\;\;\;0\;\;\;\;1 \end{array}\right]$$

In addition, the process model is related to the measurement model that describes the relationship between the state and the measurements at the current moment k as follows:

(10) $$\;Z_{k}=Hs_{t}+v_{k}$$

where $Z_{k}$ is the measurement vector, H is the measurement matrix, and $v_{k}$ is a zero-mean Gaussian measurement noise vector assumed to have covariance R, i.e., $v_{k}\sim N\left(0,R\right)$.

(11) $$\;H=\left[\begin{array}{c} 1\;0\;0\;0\\ 0\;1\;0\;0 \end{array}\right]$$

**Figure 5:**
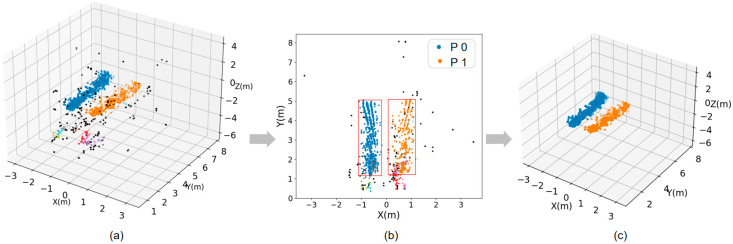
(**a**) 3D point cloud image of the original data. (**b**) Effective image after clustering. (**c**) Final point cloud cluster body image.

The time intervals adjacent to the clusters are combined with predicted values and actual measurements to obtain the best-estimated trajectories. Trajectories’ creation, updating, and deletion are heavily influenced by how well they match the clusters. Suppose a trajectory has no new matching clusters for a significant period. In that case, it is usually assumed that the entity corresponding to the trajectory is no longer in the radar’s visual range and can be deleted. In trajectory management, redundant clustering creates new empty trajectories, while redundant trajectories trigger the generation of new blank clusters. The dynamic approach to trajectory management allows for efficient tracking of the target by ensuring that the system can adapt to real-time radar data and target movement patterns in different scenarios. The trajectory to cluster matching problem is solved by employing the Hungarian algorithm. Figure 5 below shows the raw point cloud data collected by the radar and the high-quality point cloud clusters obtained by the point cloud preprocessing pipeline to prepare for the subsequent processing.

### 4.4. User Identification and Pattern-Matching

**Figure 6:**
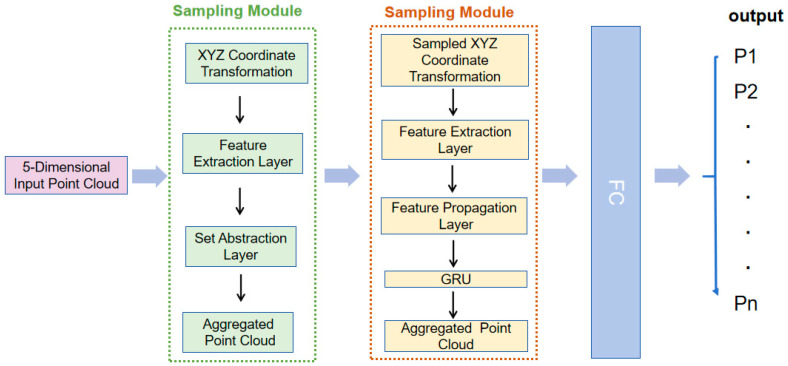
Architecture of the PGGait network.

As the 3D point cloud data generated by millimeter-wave radar typically exhibits sparsity and dispersion, transforming this data onto an image using methods like voxelization may result in a geometrically escalating computational cost for the network. As a result, it becomes difficult to process such data and perform user recognition using conventional vision methods. With this in mind, we decided to train directly using point cloud data, but choosing a network structure suitable for point cloud data takes work. By analyzing the data collected by millimeter-wave radar, we found that it contains velocity component information, which reflects features such as human limb movement and stride length. Considering these attributes, we need a network structure that can simultaneously extract spatial features during walking and temporal features during motion. Based on these considerations, we chose a PointNet-based network [32] for extracting spatial features and combined it with a lightweight GRU (Gated Recurrent Unit) network module for extracting temporal features. Together, these two form the architecture of our PGGait network. The architecture of the PGGait network is shown in Figure 6 below.

In our experiments, we conducted a comparative study to evaluate the performance of the incrementally iterative version of PointNet++ concerning the original PointNet network. The results show that the improved version using PointNet++ performs better in terms of performance. To accommodate this improvement, we extended the input layer of PointNet++ from the original three-dimensional coordinates (x, y, z) to five-dimensional coordinates (x, y, z, v, snr).

Regarding the sampling process in PointNet++, we used Farthest Point Sampling (FPS), which is a widely used point cloud sampling technique. In the XYZ Coordinate Transformation and Feature Extraction Layer, we applied FPS to select key points, en-suring the preservation of crucial information within the point cloud for subsequent processing. This process maintains point cloud uniformity by selecting the farthest points, allowing the effectively chosen key points to represent the entire point cloud. The XYZ Coordinate Transformation, Feature Extraction Layer, Set Abstraction Layer, and Aggregated Point Cloud collectively contribute to the sampling process. This methodology persists throughout the forward propagation of the entire network, gradually extracting rich feature information through the processing of the multilayer perceptron module. These components collectively ensure the effectiveness of Point-Net++ in extracting spatial features.

For the spatial coordinate data of the point cloud, we first apply the Sampling Module feature transformation for subsequent feature extraction. The data dimensions of this module are kept constant during the transfer process, with an input dimension of 3 × m and an output dimension of 3 × m, where m denotes the total number of points in the point cloud sample. We then spliced the 3 × m vector with the velocity (v) and signal-to-noise ratio (SNR) data of the point cloud to generate a 5 × m vector. Next, we input the 5 × m vector into the Multilayer Perceptron (MLP) module, i.e., MLP (64, 64), for upscaling to obtain a 64 × m vector finally. Then, we again input the upscaled vector into the Sampling Module for feature transformation and further upscaling operations to finally obtain a 1024 × m feature vector. This feature vector is processed through the pooling layer into a 1024 × 30 vector. In the multiscale module, we input this feature vector to the GRU module, which allows temporal modeling through the Feature Propagation layer and finally obtains a 128 × 30 feature vector, where ‘30’ signifies the number of time steps employed in the time-series modeling of the point cloud sequence. In the multiscale module, we input this feature vector to the GRU module, which allows temporal modeling through the Feature Propagation layer and finally obtains a 128 × 30 feature vector.

Nevertheless, due to the challenge posed by the limited capacity of the five independent gait attributes to comprehensively capture the intrinsic characteristics of gait, a fusion of these attributes becomes imperative. Finally, these five attributes are integrated by a fusion network to obtain global features of the gait point cloud, including temporal and spatial features. Finally, we use a SoftMax classifier to output the probability value of which user identity category the current point cloud data may belong to. The category with the highest probability value is the identification result determined by the network. The final recognition result is calculated as shown in Equation (12).

(12) $$\mathrm{result}\left(k\right)=\frac{e^{z_{k}}}{\sum_{n=1}^{N}e^{z_{n}}}$$

In Equation (12), result(k) is the probability of being recognized as the k individual after calculation. z denotes the vector inputted into the SoftMax layer, which contains the eigenvalues of N individuals calculated by the optimal network model. $z_{k}$ is the k element in the vector, corresponding to the k individual’s eigenvalue. Among the N probability values output from the Softmax layer, which are used to discriminate the relevance of each individual, the person number corresponding to the one with the most significant value is the final output. 

## 5. Experimental Setup

### 5.1. Hardware Platform

**Figure 7:**
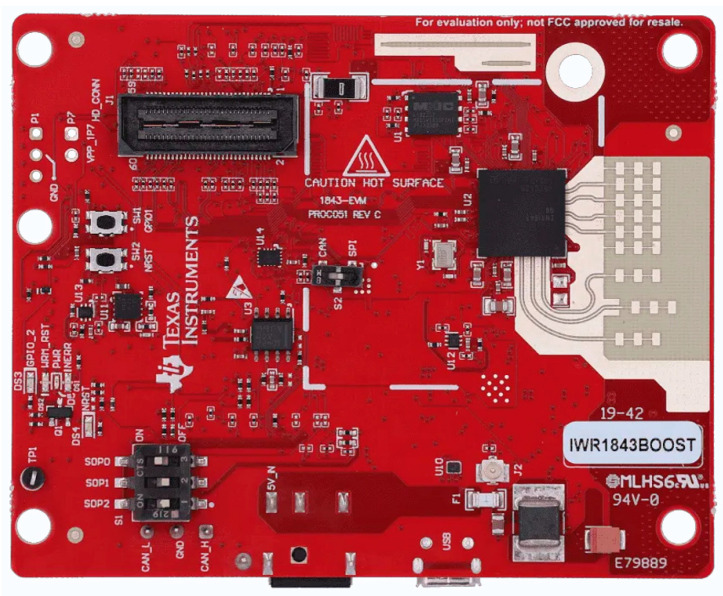
IWR1843BOOST Radar Board.

We used the IWR1843BOOST millimeter-wave radar from Texas Instruments in our experiments, as shown in Figure 7. The device encompasses a frequency range of 77 to 81 GHz and is equipped with three transmitting and four receiving antennas. It also features a single system incorporating a built-in Phase-Locked Loop (PLL) and an Analog-to-Digital Converter (ADC). The IWR1843BOOST incorporates a DSP subsystem housing a high-performance C674x DSP from Texas Instruments for radar signal processing. Furthermore, the device integrates an ARM R4F-based processor subsystem responsible for front-end configuration, control, and calibration. We take advantage of multiple antennas in order to recognize different users in complex environments. Therefore, in terms of parameter configuration, we chose a higher distance resolution setup to capture gait-related information accurately. The distance resolution for this experiment was 0.044 m, the maximum detection distance of the radar was 9 m, and the velocity resolution was 0.13 m/s. As shown in Figure 7, the radar board emitted signals to the user to collect its reflection data. Then, the collected raw data were transferred to a personal computer via a USB data cable for further analysis. The laptop used for the experiment was equipped with an Intel Core i7-8750H CPU@2.20 GHz.

### 5.2. Network Training

**Figure 8:**
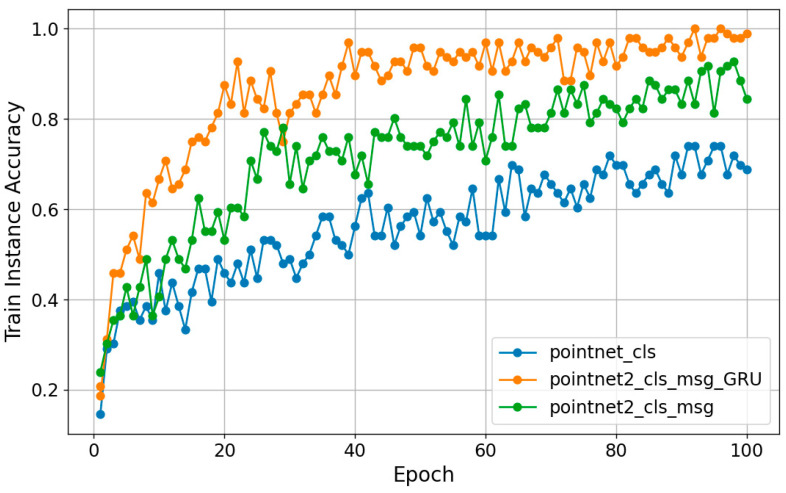
Comparison of training results of different models.

Regarding model selection, we initially adopted the PointNet network [31] as the foundational model and conducted a series of comparative experiments. These experiments encompassed PointNet_cls, PointNet2_cls_msg, and models with corresponding improvements. These experiments were conducted with the same size dataset and the same number of iterations to ensure that the selected models could achieve the best results under the same conditions. Figure 8 shows the training results.

The experimental results show that the comprehensively improved model exhibits better recognition under the same conditions. For this gait recognition task, we set the Batch size to 16 and use Adam as the optimization function, and the learning rate is set to 1 × 10^−4^, which is gradually reduced. Throughout the training process, we use PyTorch 2.0.1 and Python 3.10 to implement the functions of each module with the following configurations: an Intel Core i7-8750H CPU and an NVIDIA GeForce GTX 1050Ti. Memory size: 20GB DDR4 RAM. The entire training session lasts about 90 min or less.

### 5.3. Data Collection

**Figure 9:**
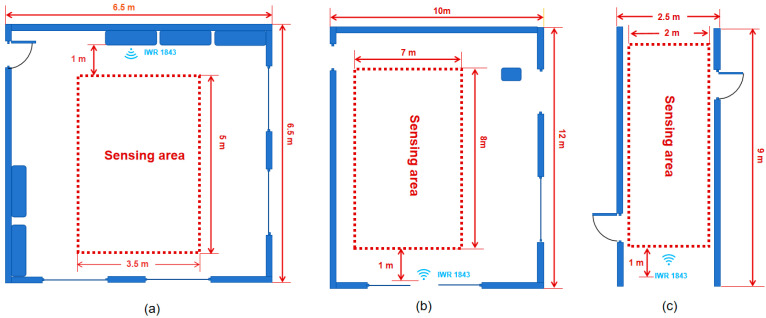
Real scene visualization. (**a**) Laboratory. (**b**) Hall. (**c**) Corridor.

In order to verify the feasibility and validity of the PGGait system, we performed actual scene data acquisition and visualized these data, as shown in Figure 9a–c. In the figures, we marked the data acquisition areas with dashed lines. The dimensions of the acquisition areas for these three scenes are 3.5 m × 5 m, 7 m × 8 m, and 2 m × 8 m. Scene 1 shows the data acquisition screen in a laboratory environment, Scene 2 shows the data acquisition screen in a lobby environment, and Scene 3 shows a corridor that is about 2.5 m wide. However, it should be noted that objects, such as iron doors and glass, on both sides of the corridor may cause unwanted reflections from the radar. Thus, there are some challenges in conducting experimental evaluations in this environment. 

**Figure 10:**
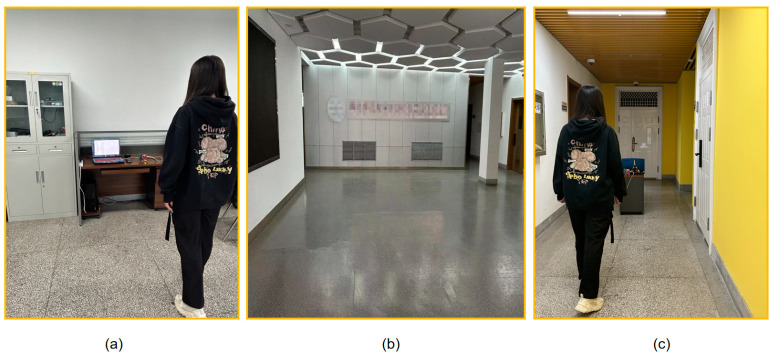
Real scenario. (**a**) Laboratory. (**b**) Hall. (**c**) Corridor.

Figure 10 below shows the data acquisition in a real scenario.

**Figure 11:**
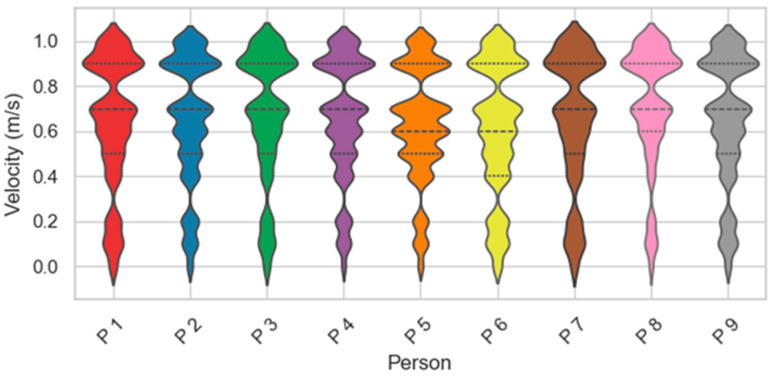
Distribution of walking speeds for different people.

In our experiments, we used a commercial radar device to collect point-cloud gait data from nine volunteers in single and two-person scenarios. These subjects consisted of four females and five males, with an age distribution between 18 and 35 years old, weight between 45 kg and 110 kg, and height ranging from 155 cm to 185 cm. Due to the millimeter-wave radar hardware’s horizontal and elevation angle constraints, we positioned the radar at a height of 0.85 m. We maintained a distance of at least 1 m to cover the range of motion of the human body adequately. During data collection, we categorized walking paths into two types: radial straight routes and non-radial routes. We accumulated about 36,000+ frames of data covering both single and two-person scenarios. The differences in limb swing amplitude and stride length of different subjects resulted in different walking speeds. These differences are the basis for performing gait recognition, so we used violin plots better to characterize the differences in walking speeds between users, as shown in Figure 11.

## 6. Evaluation

After collecting the appropriate datasets, we will evaluate the overall performance of PGGait by evaluating it in different settings and comparing it with other state-of-the-art work.

### 6.1. User Identification Performance

**Figure 12:**
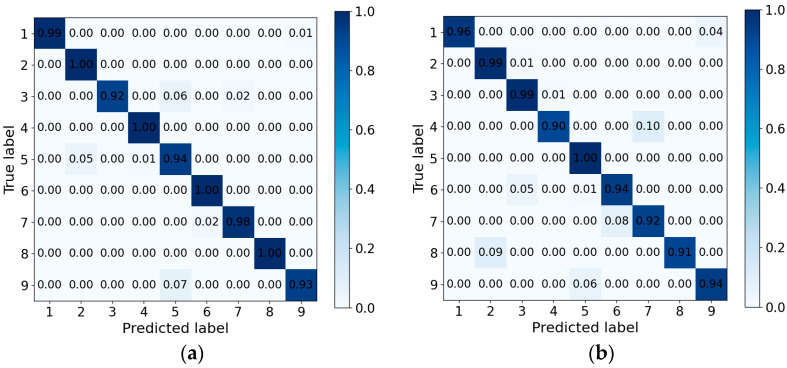
Confusion matrix recognition rate under radial path. (**a**) single user. (**b**) two persons.

We first evaluate the recognition performance of PGGait in the single-user case. As shown in Figure 12a, we demonstrate the confusion matrix for single-user recognition under radial routes. Of particular note, the average recognition accuracy is an impressive 96.75%. The lowest recognition accuracy among all participants was 92.60%. In addition, as shown in Figure 12b, we also examined the recognition accuracy in the two-person case under a radial route, and the results showed that the average accuracy reached 94.30%. In comparison, the lowest recognition rate was still as high as 91.45%. This set of recognition results validates the excellent performance of the PGGait system in recognizing single users and two-person walking situations with high accuracy.

### 6.2. Impact of Different Data Partition Ratios

**Figure 13:**
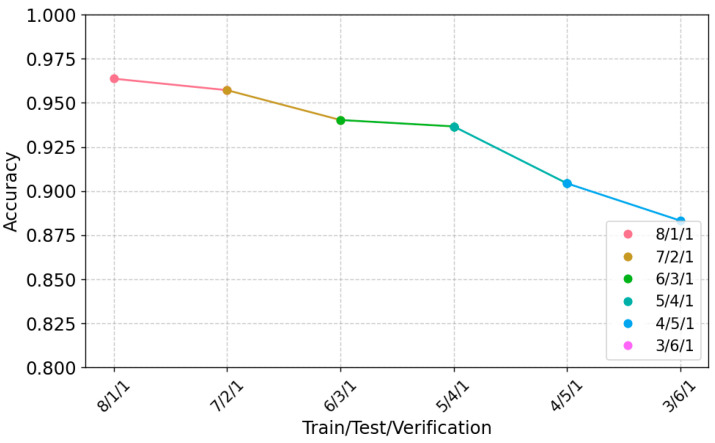
Effect of different data partition ratios.

By varying the training, test, and validation data ratio, we evaluated the performance of PGGait at different ratios. In a single-user scenario, we considered various data segmentation ratios and presented the results in Figure 13. These experimental results show that PGGait exhibits strong recognition performance under different data segmentation ratios. Our model performs well, with an average recognition accuracy higher than 93%. Of particular note, the performance is even better when the amount of training data is greater than or equal to the amount of test data. Specifically, our model maintains a recognition accuracy of about 95% when the data training and testing split ratios are above 5:4, demonstrating that PGGait achieves high performance without requiring a large amount of data.

### 6.3. Impact of Different Environments

**Figure 14:**
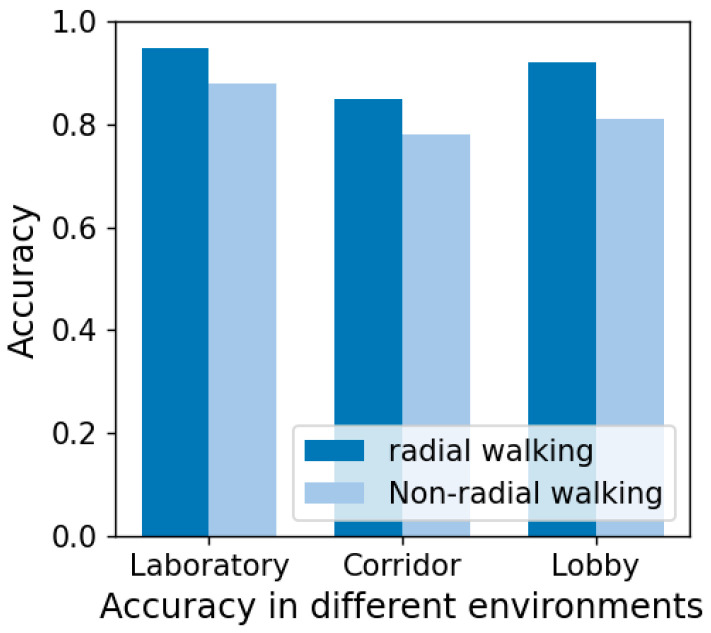
Accuracy of gait recognition obtained in three scenarios.

A series of experiments were undertaken to assess the system’s robustness across diverse environments. This involved acquiring and validating gait point cloud data from nine volunteers in three distinct settings: a laboratory, corridor, and hall. As shown in Figure 14, the experimental results indicate that the performance of the PGGait system is virtually unaffected by environmental changes. This excellent robustness is attributed to the signal processing methods employed by the millimeter-wave radar during data acquisition and the specially designed point cloud preprocessing flow, including methods such as denoising and SNR threshold filtering. These methods effectively eliminate point cloud data from interfering objects in complex environments, such as walls, ceilings, glass, doors, and chairs, resulting in high-quality point cloud data that minimizes the impact of environmental factors on recognition performance. As a result, millimeter-wave radar-based gait recognition techniques show higher stability and applicability in processing data from different environments compared to WiFi-based gait recognition methods.

### 6.4. Effects of Different Walking Directions

**Figure 15:**
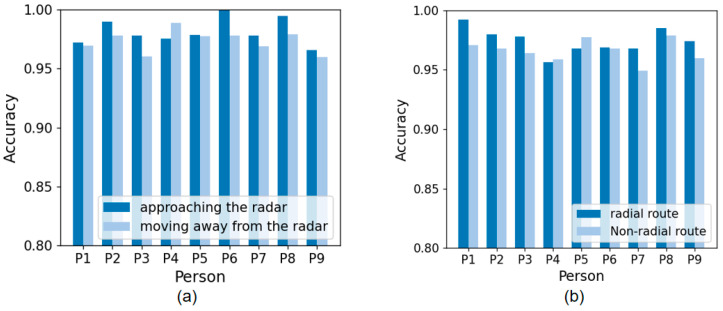
(**a**) Accuracy of walking towards and away from the radar. (**b**) Accuracy of different walking routes.

In order to verify the performance difference of the system in different walking directions, we conducted a series of experiments, firstly testing the system in different walking directions of a single user on the radial path of the radar, including two directions toward the radar and away from the radar. The results show that the difference in final recognition accuracy between the two directions under a single walking direction is only 3%, as shown in Figure 15a. We also conducted experiments in which data from a single direction toward the radar was trained and then tested in the direction away from the radar. However, the experimental results showed that meaningful data could not be obtained in this case. Therefore, combining data from both directions is required for practical applications. Finally, after comparing the test results of the radial radar path and the non-radial path, it is found that the recognition rate under the non-radial path is slightly lower than that under the radial path, as shown in Figure 15b.

### 6.5. Comparison with Recent Research Work

We compare the recognition accuracy of the PGGait system with other recent research works, including the solutions of mmGaitNet [25], Wu et al. [33], MTPGait [26], Xia et al. [34], and Pegoraro [35], and the results of the comparisons are shown in Table 1. mmGaitNet’s research uses a dual radar technique, which shows good recognition of walks on radial paths. However, the recognition accuracy on non-radial paths drops significantly to 45%. This suggests that the algorithm in this work has challenges adapting to the point cloud data under non-radial paths. Wu et al.’s study addresses the issues of multipath interference and complex walking in the scene mainly through a specialized CNN network but ends up with a limited recognition result of 75%. MTPGait’s work achieves a relatively high recognition accuracy in single and two-person scenarios, thanks to the multi-dimensional data input and the combination of the CNN + LSTM network. However, the method requires a large amount of training data to achieve these results. Pegoraro’s study uses micro-Doppler techniques but needs more adaptability and a large amount of training data, which leads to a significant drop in accuracy in other environments.

Considering the above results, compared to existing work, the PGGait system is still able to achieve high recognition accuracy with relatively small datasets by improving the quality of the point cloud data and analyzing the gait data in conjunction with our designed network for extracting the spatio-temporal information, despite the limited data acquisition equipment. In addition, the PGGait system can maintain high recognition accuracy in different environments, including radial and non-radial paths.

### 6.6. Ablation Experiment

To validate the contribution of the innovative elements proposed in our study to gait recognition performance, we conducted a series of ablation experiments, selecting Pointnet2 as the baseline network version according to Section 5.2. We specifically examined two critical factors in the model: the addition of GRU modules and the application of data preprocessing. In Table 2 below, we compared the accuracy under different configurations, with particular emphasis on assessing the impact of adding GRU modules and applying data preprocessing on final recognition accuracy.

Initially, we established a baseline network version based on PointNet2_cls_msg. Subsequently, GRU modules were separately integrated into the model, and data preprocessing was applied. To ensure experimental reproducibility, we ran the experiments multiple times to obtain reliable results. The experimental outcomes indicate that, among various configurations, those involving the addition of GRU modules and data preprocessing exhibited optimal performance in terms of both accuracy (Best) and accuracy (Avg), achieving an accuracy of approximately 94.74%.

The results of the ablation experiment strongly affirm the effectiveness of our proposed approach. In the experimental configurations, the highest gait recognition accuracy was achieved by incorporating data preprocessing operations and introducing GRU modules into the baseline network, providing robust support for our innovative contributions. We observed a significant positive impact on the model’s performance by adding GRU modules and applying data preprocessing. This suggests that through the introduction of temporal modeling and data preprocessing techniques, our model is better equipped to capture spatiotemporal information, thereby enhancing gait recognition accuracy.

### 6.7. Discussion

The PGGait user gait recognition system first uses raw point cloud data collected by millimeter-wave radar, which is then passed into a specially designed point cloud preprocessing process to obtain high-quality point cloud data that maximally eliminates interferences caused by complex factors such as the environment. Next, denoising and clustering optimization are carried out in the clustering and tracking stage to obtain high-quality point cloud clusters for subsequent work. At the same time, the input of a multidimensional point cloud is formed by expanding the original point cloud data to increase the speed and signal-to-noise ratio. In addition, the original PointNet++ network module was modified to add a GRU module for multiscale spatio-temporal feature extraction, which fuses spatial and temporal features to significantly improve the model’s accuracy, thus enhancing the overall performance. The experimental results in various scenarios show that the PGGait user identification system can achieve higher recognition accuracy than other recent work. For future work, we plan to investigate the distinctions among gaits with similar body postures and address variances in gait patterns exhibited by users with mobility challenges. Simultaneously, to further enhance the overall system’s recognition performance, we will consider factors related to experimental equipment. Utilizing configurations characterized by high bandwidth and multiple transmit-receive antennas can enhance the accuracy of data collection. Moreover, refining target filtering, clustering, and tracking algorithms can further elevate data precision and recognition accuracy at the data processing stage. It is essential to holistically consider factors at each stage to improve the overall final recognition accuracy.

## 7. Conclusions

In this study, we proposed a gait recognition system based on millimeter-wave radar spatio-temporal sensing of multidimensional point clouds named PGGait. The system utilizes a specially designed point cloud preprocessing pipeline to extract high-quality point clouds. It innovatively inputs multidimensional point cloud data into a neural network specially designed to extract spatial and temporal features. We implemented the system on a commercial 77 GHz millimeter-wave radar. We conducted robustness experiments, encompassing various user walking directions, different data segmentation ratios, and diverse experimental scenarios, including laboratory, lobby, and corridor settings. The experiments involved nine users exhibiting variations in height, weight, gender, and age. The experimental results show that the system achieves up to 96.75% accuracy in single-user recognition and 94.30% in the two-user scenario. These results further validate the robustness and effectiveness of our proposed system. Future research directions include expanding the application of more radar devices and introducing a multimodal approach to solve the occlusion problem between users to improve the performance of multiuser recognition further. This will help extend the applicability of our system in real-world scenarios and expand its potential in various application areas. Our study provides a solid foundation for applying millimeter-wave radar in user gait recognition and provides valuable guidance and reference for future research and practical applications.

## Figures and Tables

**Table 1:** Comparison with recent research work.

Method	Data Format	Experimental Scenarios	Number of Experimenters			
			1 (Radial)	1 (Non-Radial)	2 (Radial)	2 (Non-Radial)
mmGaitNet [25]	Point Clouds (two radar)	2	86.00%	NA	85.00%	45%
Wu et al. [33]	Gait Spectrum	1	NA	68.88%	NA	75.51%
MTPGait [26]	Point Clouds (single radar)	3	94.70%	92.70%	89.90%	87.90%
Xia et al. [34]	Point Clouds (single radar)	1	85.8%	87.00%	NA	NA
Pegoraro [35]	Micro-Doppler	1	95.76%	NA	93.45%	NA
PGGait (ours)	Point Clouds (single radar)	3	96.75%	94.74%	94.30%	90.32%

**Table 2:** Impact of different modules on performance.

GRU	Preprocessing	Accuracy (Best)	Accuracy (Avg)
×	×	78.32%	67.34%
√	×	90.63%	84.13%
×	√	85.63%	80.73%
√	√	97.75%	94.74%

## Data Availability

Data are contained within the article.
